# Placing the focus on perfectionism in female adolescent anorexia nervosa: three cases of the use of augmented Maudsley Family Based Treatment

**DOI:** 10.1186/2050-2974-1-S1-O28

**Published:** 2013-11-14

**Authors:** Kim Hurst

**Affiliations:** 1 Eating Disorder Program (EDP) CYMHS, Australia

## 

Although Family Based Treatment (FBT) appears to be demonstrating positive treatment outcomes in terms of weight restoration and menstruation, maintaining psychological factors such as perfectionism have largely been overlooked. At the conclusion of FBT it has been reported that approximately 60% of cases receive follow-up or engage in supplementary psychological treatment (Lock et al., 2006; Lock et al., 2010). Perfectionism is one of the maladaptive cognitive patterns that seems important to the onset and/or maintenance of Anorexia Nervosa (AN), further it has been linked to treatment resistance and relapse (Franco-Paredes, et al., 2005). By concentrating on those maintaining mechanisms that appear to be necessary for the AN to persist, it has been hypothesised that reducing perfectionism would improve AN symptoms and rates of recovery. It is therefore timely to compliment FBT, which is commonly considered to be the most efficacious treatment of AN in adolescents, with a therapy that specifically addresses perfectionism. The purpose of the paper is to introduce a cognitive behavioural treatment component focused on perfectionistic thinking and related maladaptive thought patterns, which has been used to augment FBT for AN. By adding components to specifically address perfectionism using CBT techniques, outcomes for adolescents in FBT may be substantially improved; such an approach is described with illustrative examples of its effectiveness with three adolescent females diagnosed with AN.

This abstract was presented in the **Disordered Eating – Characteristics & Treatment** stream of the 2013 ANZAED Conference.

